# Herbal Medications in Endodontics and Its Application—A Review of Literature

**DOI:** 10.3390/ma15093111

**Published:** 2022-04-25

**Authors:** Mohmed Isaqali Karobari, Abdul Habeeb Adil, Ali A. Assiry, Syed Nahid Basheer, Tahir Yusuf Noorani, Ajinkya M. Pawar, Anand Marya, Pietro Messina, Giuseppe Alessandro Scardina

**Affiliations:** 1 Conservative Dentistry Unit, School of Dental Sciences, Universiti Sains Malaysia, Health Campus, Kubang Kerian, Kota Bharu 16150, Kelantan, Malaysia; dentaltahir@yahoo.com; 2 Department of Restorative Dentistry & Endodontics, Faculty of Dentistry, University of Puthisastra, Phnom Penh 12211, Cambodia; 3 Department of Conservative Dentistry & Endodontics, Saveetha Dental College and Hospitals, Saveetha Institute of Medical and Technical Sciences, Chennai 600077, India; 4 Dental Public Health Unit, School of Dental Sciences, Universiti Sains Malaysia, Health Campus, Kubang Kerian, Kota Bharu 16150, Kelantan, Malaysia; drhabeebadil@gmail.com; 5 Preventive Dental Science Department, Faculty of Dentistry, Najran University, Najran 55461, Saudi Arabia; assirypedo@gmail.com; 6 Department of Restorative Dental Sciences, College of Dentistry, Jazan University, Jazan 45142, Saudi Arabia; syednahidbasheer@gmail.com; 7 Department of Conservative Dentistry and Endodontics, Nair Hospital Dental College, Mumbai 400008, India; ajinkya@drpawars.com; 8 Department of Orthodontics, Faculty of Dentistry, University of Puthisastra, Phnom Penh 12211, Cambodia; amarya@puthisastra.edu.kh; 9 Department of Surgical, Oncological and Stomatological Disciplines, University of Palermo, 90133 Palermo, Italy; pietro.messina01@unipa.it

**Keywords:** herbal medications, endodontics application, root canal, natural products

## Abstract

Herbal products are gaining popularity in dental and medical practice nowadays due to their biocompatibility, higher antimicrobial activity, antioxidant and anti-inflammatory properties. Herbal medicine has experienced rapid growth in recent years due to its beneficial properties, ease of availability, and lack of side effects. As pathogenic bacteria become more resistant to antibiotics and chemotherapeutic agents, researchers are becoming more interested in alternative products and treatment choices for oral diseases. As a result, natural phytochemicals separated from plants and utilized in traditional medicine are suitable substitutes for synthetic chemicals. The aim of this review article is to list and understand several herbal alternatives that are currently accessible for use as efficient endodontic medicaments. The herbal products used in endodontics have several advantages, including safety, ease of use, increased storability, low cost, and a lack of microbial tolerance. However, preclinical and clinical testing and interactions with other materials and adverse effects are required for these herbal products.

## 1. Introduction

Herbal products are gaining popularity in dental and medical practice nowadays due to their biocompatibility, higher antimicrobial activity, antioxidant and anti-inflammatory properties [1]. Herbal medicine is defined by the World Health Organization as a plant-originated preparation or material that includes processed or raw components from one or more plants that have medicinal properties [2]. The use of herbal alternatives for root canal treatment is becoming more popular. “Phytotherapy, Phytomedicine, or Ethnopharmacology” is the term for using herbals to treat various ailments. Herbal medicine has experienced rapid growth in recent years due to its beneficial properties, ease of availability, and lack side effects [3].

As pathogenic bacteria become more resistant to antibiotics and chemotherapeutic agents, researchers are becoming more interested in alternative products and treatment choices for oral diseases. As a result, natural phytochemicals separated from plants and utilized in traditional medicine are suitable substitutes for synthetic chemicals [4]. Herbs could be a good substitute for conventional treatments for oral health issues, but there is a lack of knowledge about their effect on oral tissues, mechanisms of action, and side effects. As a result, more research is needed to investigate these traditional medicines [5].

Biofilms are microbial communities that adhere to a specific surface and are shielded by a polymeric matrix [6]. The infected root canal system contains all three elements required to form a microbial community: solid residue, fluid streams, and microorganisms [7]. Herbal medicines are now being incorporated into toothpaste to help prevent dental caries. Polyphenols’ anti-cariogenic properties are primarily due to a direct effect on *S. mutans*, and it interacts with microbial membrane proteins to prevent bacterial cells from adhering to the tooth surface [8]. The promise of using non-ionizing radiation diagnostic tests in dentistry, as well as the challenges associated with its application, has prompted scientific research in this area to produce intriguing discoveries that bode well for the future. According to a large body of evidence gathered in every department of dentistry from the implementation of these diagnostic examinations, magnetic resonance imaging (MRI) and ultrasonic imaging represent the most exciting developments in this field [9].

Herbs are primarily used in endodontics for root canal disinfection. Due to the adverse effects of most synthetic intracanal medications, there has been an increase in research into herbal irrigants. The bacteria *E. faecalis* is the most common cause of root canal treatment failure [10]. The unique behavior of microorganisms, resulting from their organization in complex microbial communities, necessitates extra caution when treating root canal infectious diseases [11]. The thorough cleaning of the root canal system is among the most essential goals of endodontic treatment, free of microbes and debris, and it also involves removing infected tissue from within the root canal in order to seal it with a microbial tight filling and to prevent infection of the peri-radicular tissues and aid in their healing [12,13]. This can be accomplished by chemically treating the root canal system. This treatment includes biomechanical preparation, irrigation, and medication administered between appointments [14].

However, large portions of the root canal are left untouched during instrumentation, resulting in failure of the root canal treatment. Irrigation aids in the proper disinfection and eradication of infected microbes. NaOCl, chlorhexidine, hydrogen peroxide, ethylenediaminetetraacetic acid (EDTA), citric acid, and other irritants are used [15]. The advantages of employing herbal alternatives include easy availability, prolonged storage life, cost-effectiveness, minimal toxicity, and the lack of microbial resistance recorded so far [16]. Therefore, the aim of this review article is to list and understand several herbal alternatives that are currently accessible for use as efficient endodontic medicaments.

## 2. Methodology

The literature search was conducted indefinitely using the Google scholar, MEDLINE, PubMed databases (Table 1). Reference lists of potentially relevant articles and review articles in the English language were screened. The following keywords were used in the search strategy: Herbal Intervention in Endodontics, Herbal Medications used in Endodontics, Natural Products used in Endodontics. Only studies on herbal products in endodontics were considered; studies on other dental specialties were not. Some of the articles were labelled as reviews. Two reviewers (AHA and MIK) screened the articles.

## 3. Results

**Figure 1:**
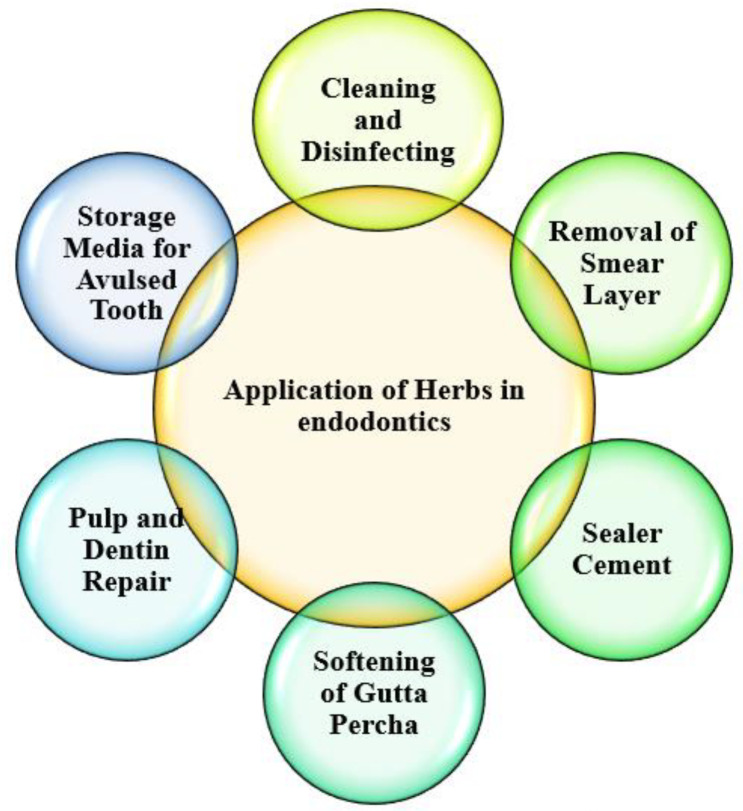
Application of herbs in endodontics.

The search yielded 2023 articles, 1935 of which were eliminated due to non-compliance with the criteria of inclusion of the articles. Eighty-eight searched articles were selected as they met the inclusion criteria. Pulp and dentin repair, cleaning and disinfection, removal of smear layer, sealer cement for lubricating and aiding in the bonding of gutta-percha obturation material, elimination of obturation material by dissolving and softening it, and avulsed teeth storing media are all examples of herbal products used in endodontics. The different uses of herbal medications in endodontics are shown in Figure 1.

**Figure 2:**
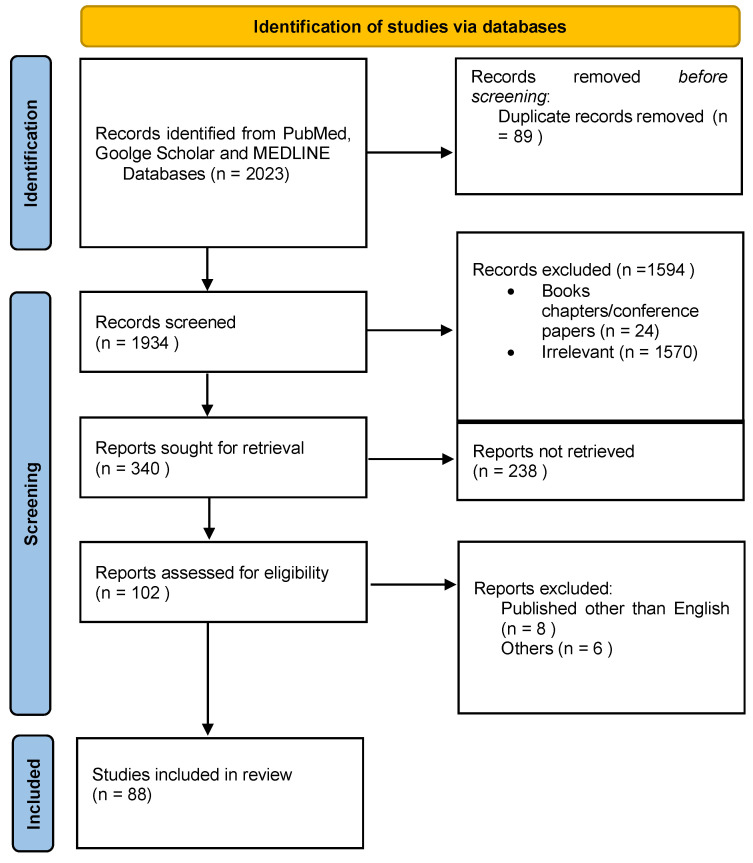
PRISMA flowchart showing the selection process of articles retrieved from different web sources.

Figure 2 depicts the selection criteria as it follows the PRISMA (Preferred Reporting Items for Systematic Reviews and Meta-Analyses) guidelines. These 88 articles were examined for the current study based on the quality of the research studies.

## 4. Discussion

Over the past decade, there has been a significant surge of attention in preparations derived from medicinal plants. Many plants have been mentioned in the literature as a potential source of new endodontic therapies. In dentistry, phytomedicine has been utilized as an antibiotic, endodontic irrigant, anti-inflammatory, sedative, and analgesic [17]. Because most commercial intracanal medications cause cytotoxicity and are unable to eliminate microorganisms from dentinal tubules, biologic medications produced from natural plants have become popular in recent years [18].

### 4.1. Pulp and Dentin Repair

Pulp capping can be done with various materials to induce tissue repair dentinogenesis across the exposed pulp. The material chosen is calcium hydroxide, which is widely accepted. Although many researchers have advocated direct bonding of pulp exposure, it is still controversial [19]. Natural herbs could also be a feasible pulp capping option. According to a group of researchers, the natural product called baicalin, a flavonoid extracted from the plant’s root, enhanced the angiogenesis and odontoblastic differentiation of Human Dental Pulp Cells by promoting angiogenic factors, mineralization of alkaline phosphatase activity, and morphogenetic protein expression [20]. Lower amounts of ascorbic acid increased chondrogenesis and osteogenesis while decreased adipogenesis in stem cells from human exfoliated deciduous tooth. Ascorbic acid increased the release of growth factors, anti-inflammatory cytokines, and bone metabolism-related factors. Stem cells from human exfoliated deciduous tooth treated with ascorbic acid may offer novel therapy options for tooth and bone regeneration. It may be used in pediatric pulp capping, apexogenesis, cleft lip, and other congenital abnormalities [21]. Various studies related to the use of herbal medicine for the pulp and dentin repair used in endodontics are illustrated in Table 2

### 4.2. Cleaning and Disinfection

Irrigants have used a variety of synthetic antimicrobial agents to clean and disinfect root canals over the years. Because of rising resistance to antibiotics to certain antimicrobials, as well as the toxic and unpleasant side effects of a few prevalent antibacterial agents, there is a need for alternative agents that are nontoxic, inexpensive, and effective. Natural plant extracts have been discovered to clean and disinfect root canal irrigants [29] effectively. An endodontic irrigant should have the following properties: it should be nontoxic systemically, it should not cause an anaphylactic reaction, it should not harm periodontal tissues, it should have a robust antibacterial spectrum, it must be able to solubilize or prevent the development of a smear layer, as well as disintegrate the necrotic pulp tissue and deactivate endotoxins [15].

Sodium hypochlorite is amongst the most commonly utilized root canal irrigants because of its ability to kill a wide range of bacteria. Allergic potential, tissue toxicity, an unpleasant taste, and the failure to eradicate the smear layer are some of its disadvantages [32]. Because of its broad-spectrum antibacterial activity, chlorhexidine is another commonly used antimicrobial drug for irrigating root canals, disinfect infected root canals and biocompatibility [33]. However, it lacks tissue disintegrating properties and has some adverse side effects, such as discoloring the teeth, causing oral dryness, and even causing a burning sensation in the mouth [34]. Table 3 illustrates the studies related to herbal medicine for cleaning and disinfecting root canals.

### 4.3. Removal of Smear Layer

Because microorganisms are found in all sections of the root canal system, notably in lateral canals, anastomoses, and dentinal tubules, mechanical preparation alone is ineffective in removing pulpal remains and microorganisms from root canals [47]. To eliminate the smear layer, chelating chemicals such as citric acid, ethylenediaminetetraacetic acid (EDTA), and maleic acid have been utilized. However, dentinal erosions, decreases in dentin microhardness, biocompatibility issues, and allergic reactions have all been reported as side effects of employing these chemical formulations [48,49]. The most commonly used smear layer remover is EDTA, which aids root canal cleaning by interacting with inorganic debris. Its contact with calcium ions in dentine promotes chelation of calcium, resulting in dentine demineralization in 5 min at 20–30 lm depths [50]. Table 4 shows the studies related to the use of herbal medicine for removal of smear layer.

### 4.4. Sealer Cement for Lubricating and Aiding in the Bonding of Gutta-Percha Obturation Material

The purpose of root canal therapy is to remove bacterial infections. Because the microorganisms persist in the dentinal tubules and root canal after the instrumentation, only chemo-mechanical canal preparation may not achieve the goal of root canal treatment [59]. It is desirable to employ sealers with the strong sealing ability and antimicrobial properties [60]. Because endodontic medications come into contact with tissues peri-apically, the sealers must be minimally cytotoxic [61]. Researchers are looking for natural alternatives to synthetic pharmaceuticals due to the ongoing rise in antibiotic resistance strains and adverse effects induced by synthetic drugs. Even though herbs have a wide range of applications in medicine, there have been few investigations in dentistry. Table 5 demonstrates the studies related to herbal medicine in combination with commercially available endodontic sealers.

### 4.5. Removal of Obturation Material by Softening and Dissolving

When nonsurgical root canal therapy fails, an endodontically failed tooth may require treatment. The failure of nonsurgical endodontic therapy has been linked to several factors, including insufficient root canal system debridement and procedural mistakes. Gutta-percha is the most often used obturation substance [68]. A great deal of effort has developed a therapeutically viable procedure for removing this debris from the root canal. Thermal, mechanical, chemical, or a mix of these approaches are employed to remove the gutta-percha [69]. Although chemicals have been used for years to remove gutta-percha, the most successful chemicals are toxic or otherwise dangerous. Benzene, for example, has good solvent properties but is extremely flammable, with a flash point ranging from 0 to 12 degrees Celsius [70]. One of the most efficient solvents is chloroform. Alternative materials such as xylol, halothane, and tetrachloroethylene have arisen due to their possible carcinogenicity [71]. In dentistry, essential oils are utilized as gutta-percha solvents, smear layer removers, and antibacterial agents [71]. The intrinsic safety of essential oils encourages them to be searched for alternate solvents to chloroform. Table 6 illustrates the studies related to herbal medication for softening and dissolving gutta-percha.

### 4.6. Avulsed Teeth Storing Media

Traumatic injuries to the anterior teeth are most common in children aged 7 to 10, with tooth evulsion occurring in 0.5 per cent to 16 per cent of cases. One of the most severe types of dental trauma is avulsion injury [77]. The vitality of the periodontal ligament (PDL) cells persisting on the root surface, the quality of the root cementum, and minimum bacterial contamination determine the prognosis of a transplanted tooth and its preservation on the dental arch for the maximum time duration, which is directly connected to extra-alveolar time, storage form after avulsion, and root surface modifications [78]. Immediate tooth replantation improves PDL repair and dramatically minimizes the risk of root resorption. As a result, cutting the time between trauma and tooth replantation as short as feasible and keeping the avulsed tooth in a suitable transport medium might reduce the adverse effects of the extrabuccal period on the root surface and improve the prognosis significantly [79]. Numerous research has been conducted on various storage media that may aid in the vitality of periodontal ligament cells [80]. However, none of the currently employed media can meet all the ideal parameters for preserving cell viability. As a result, the search for an appropriate storage medium continues. Herbal products are readily available for the trauma site may benefit adequate storage capacity and cell viability [81]. Table 7 shows the studies related to herbal medicine for the storage of avulsed teeth.

## 5. Future Implications and Recommendations

Herbs and other natural remedies are employed in endodontics to minimize inflammation and alleviate tissue irritation. As mentioned above and their products, the substances found in the herbs have been demonstrated to have antimicrobial activity against oral bacteria such as *S. mutans*, *Candida albicans*, and other pathogens in research. However, preclinical and clinical testing and interactions with other materials and adverse effects are required for these herbal products. There is still much room to learn about nature and its products to use in our profession. These herbal medications can be advantageous in nations where the bulk of the population cannot afford pricey therapies due to their advantages. However, further research is needed before being used in standard endodontic treatment.

## 6. Conclusions

Herbal products used in endodontics have several advantages, including safety, ease of use, increased storability, low cost, and a lack of microbial tolerance. Today is the era of scientific proof medicine, which means that any pharmaceutical intended for human use must undergo comprehensive in vitro and in vivo testing. Herbal products appear promising in vitro, but biocompatibility and safety must be evaluated in preclinical and clinical research before they can be definitively suggested to be used in endodontics. Herbs are generally harmless when appropriately used, but they can be hazardous if taken incorrectly.

## Figures and Tables

**Table 1:** Information of sources and Search strategies using MeSH keywords.

Database	Search Strategies	Results
PubMed	((((((((((((Herbal[Title/Abstract]) OR (Natural[Title/Abstract])) OR (Herbal products[Title/Abstract])) OR (Natural products[Title/Abstract])) OR (Herbal products in endodontics[Title/Abstract])) OR (Natural products in endodontics[Title/Abstract])) OR (Herbal intervention in endodontics[Title/Abstract])) OR (Root canal[Title/Abstract])) OR (Dental applications[Title/Abstract])) OR (Herbal endodontics[Title/Abstract])) AND (((((((Herbal medication[Title/Abstract]) OR (Endodontics applications[Title/Abstract])) OR (Herbal medications in endodontics[Title/Abstract])) OR (Herbal endodontics[Title/Abstract]))	586
Google Scholar	Herbal OR Natural OR Endodontics OR Root canal OR Application OR Herbal products OR Natural products OR Root canal system OR Endodontics OR Herbal endodontics OR Herbal medications OR Natural medications OR Herbal intervention AND Herbal products in endodontics OR Application of herbal medicine in endodontics OR Natural products used in endodontics	1048
MEDLINE	Herbal OR Natural OR Endodontics OR Root canal OR Application OR Herbal products OR Natural products OR Root canal system OR Endodontics OR Herbal endodontics OR Herbal medications OR Natural medications OR Herbal intervention AND Herbal products in endodontics OR Application of herbal medicine in endodontics OR Natural products used in endodontics	389
Total		2023

**Table 2:** Studies related to the use of herbal medicine for pulp and dentin repair.

Herbal Material	Characteristics	Reference
Propolis	➢ Aids in the formation of hard tissue bridges ➢ Ability to stimulate multiple enzyme systems, circulation, cell metabolism, and collagen formation	[22,23]
Baicalin	➢ Acceptable and effective pulp agent ➢ It has a tremendous clinical pledge in the treatment of acute deep caries ➢ Effects better than Ca (OH)_2_ with direct pulp capping	[24,25]
Acemannan	➢ Vital pulp therapy in primary teeth ➢ Ability to form reparative dentin ➢ Suitable for direct pulp capping	[26]
Galla Chinensis Extract	➢ Promising direct pulp capping material ➢ Less biological pulp response in comparison to MTA	[27]
Nigella stevia	➢ Pulp capping agent for deciduous teeth ➢ It has an anti-inflammatory effect ➢ It retains pulpal vitality after application	[28]
Genipin	➢ Genipin-enhanced alkaline phosphatase action ➢ Odontogenic marker expression and mineralized nodulation help trigger human dental pulp cells odontogenic differentiation.	[29]
Green Tea Polyphenols	➢ Tooth decay-causing bacterial growth and occurrence are inhibited. ➢ Contains fluoride(natural), which may aid in the prevention of dental caries	[30,31]

**Table 3:** Studies related to the use of herbal medicine for cleaning and disinfecting root canals.

Herbal Material	Characteristics	References
Triphala	➢ Anti-oxidant property ➢ Remove smear layer ➢ Inhibit biofilm formation	[30,35]
Azadiratcha Indica (Neem)	➢ Significantly decrease the plaque index and bacterial count ➢ Antimicrobial properties against *S. mutans*, *E. faecalis*, and *Candida albicans*	[36,37]
Propolis (Bee glue)	➢ Good antimicrobial and anti-inflammatory agent ➢ Anesthetic and cytotoxic properties ➢ Antioxidant	[38]
Marticariarecutitia L (German chamomile)	➢ Anti-inflammatory, analgesic, anti-spasmodic, and sedative properties ➢ Very effective in eliminating the smear layer	[39]
Melaleuca alternifolia (Tea tree oil)	➢ Solvent action ➢ Dissolves necrotic tissue ➢ Anti-septic agent	[1,40]
Curcuma longa (Turmeric)	➢ Antioxidant and anti-inflammatory agent ➢ Exhibit phototoxic effect on bacteria ➢ Used mainly in root canal failure cases in endodontics	[41,42]
Salvadora persica (Miswak)	➢ Contain large amounts of salvadorime chloride, trimethylamine, and fluoride ➢ A significant anti-microbial effect	[43,44]
Allium sativum (Garlic)	➢ Root canal bacteria cell wall and cell membrane are destroyed ➢ It has antimicrobial properties but is not as strong as sodium hypochlorite or chlorhexidine	[32,45]
Morinda citrifolia (Indian mulberry)	➢ As a root canal irrigant, it has efficacy similar to sodium hypochlorite ➢ Effective chelating agent	[30]
Carvacrol	➢ Disruption of bacterial cell membrane ➢ Periapical tissue repair	[46]
Myrtus communis	➢ Effective agent against persistent root canal microorganisms	

**Table 4:** Studies related to the use of herbal medicine for removal of smear layer.

Herbal Material	Characteristics	References
Green tea extract	➢ Higher action against a broader range of microorganisms ➢ A good chelating agent	[51]
Neem leaf extract	➢ Presence of flavonoids and acid metabolites ➢ The highest amount of smear layer removal	[52]
Chitosan	➢ Acts on an inorganic portion of smear layer ➢ Effectively remove smear layer from apical and middle areas of root canal	[50]
Oregano extract solution	➢ Have the same effect as NaOCl in removing the smear layer ➢ Strong antibacterial	[53]
Triphala	➢ More effective in the removal of smear layer when compared to distilled water	[54]
Morinda citrifolia juice	➢ Effectively removes smear layer when used with 6% of concentration with pH 3.5 ➢ Contains mild acidic action, which has a chelating effect	[55]
Matricaria recutita extract	➢ Most commonly used herbal medicine ➢ Contains acid, which helps in removing the smear layer	[56]
Noni juice, citrus and carbonic acid juice	➢ Chelating effect better than EDTA when irrigated for 30 min ➢ Effective removal of smear layer	[57]
Salvadora persica (Miswak)	➢ Promising herbal medication for the removal of smear layer	[58]

**Table 5:** Studies related to the use of herbal medicine in combination with commercially available endodontic sealers.

Herbal Material	Characteristics	References
Licorice, Bakul and Guguchi added to the commercial root canal sealers	➢ Significant inhibition of bacterial growth ➢ Efficient antimicrobial	[62]
Amla, Miswak and Nutmeg added to the commercial sealers	➢ Rich source of bioactive compounds that possess antimicrobial properties ➢ Produces bacterial inhibitory zones	[63]
Cinnamon oil	➢ Maximum zone of bacterial inhibition ➢ Inherently antimicrobial	[64]
Moringa root	➢ Acceptable physical properties when mixed with commercially available endodontic sealers	[65]
Tamarillo skin extract	➢ Inhibition of *Enterococcus faecalis* bacterial growth	[66]
Propolis and Green tea	➢ More effective in inhibiting bacterial growth	[67]

**Table 6:** Studies related to the use of herbal medication for softening and dissolving of gutta-percha.

Herbal Material	Characteristics	References
Castor oil, Peppermint oil and Wintergreen oil	➢ Effective herbal solvents for the removal of gutta-percha ➢ Readily available and economically feasible	[72]
Clove oil, Orange oil and Eucalyptus oil	➢ Most efficient solvents to dissolve gutta-percha ➢ Less toxic	[73]
Cardamom seeds Oil and dry ginger rhizomes	➢ Enhanced ability to penetrate gutta-percha polymer ➢ oxygenated monoterpenes in increasing concentrations	[71]
Tea tree oil	➢ Exhibited dissolving potential	[74]
Turmeric	➢ Used for endodontic retreatment	[75]
Tangerine, Grapefruit, Lemon Oils and Lime	➢ The surface dissolving depth and maximum force required for piercing the spreader of 5 mm depth were discovered.	[76]

**Table 7:** Studies related to the use of herbal medication for storage of avulsed tooth.

Herbal Material	Characteristics	References
Vaccinium macrocarpon, Punicia granatum, Psidium guajava, Prunus domestica and Camellia sinensis	➢ These five herbal research media can be employed to transfer avulsed teeth effectively. ➢ They have the potential to sustain viable cells even when the extra-alveolar duration is extended, with a viability loss of 24.7 per cent from 15 min to 3 h.	[81]
Propolis	➢ The results were good for maintaining cell viability; however, root resorptions were observed, reducing its efficacy for this purpose.	[82]
Green tea extract	➢ Green tea was found to be an excellent medium for limiting infections following tooth replantation, maintaining PDL cell viability, and lowering root resorption and ankylosis, with 90 per cent cell viability maintained for up to 24 h.	[83]
Morus rubra (red mulberry)	➢ The ability of red mulberry to sustain the viability of PDL cells was improved when teeth were stored in it for up to 12 h.	[84]
Coconut water	➢ The natural and sterile storage medium ➢ Medium with good biological features and easy access that could be beneficial for its indication	[85]
Aloe vera	➢ When compared to other experimental storage media, PDL cells were significantly maintained by the aloe Vera ➢ Aloe Vera 10 percent, 30 percent, and 50 percent may be advised as an appropriate storage medium for avulsed teeth.	[86]
Rice water	➢ After 30 min, rice water contains a large number of live periodontal ligament cells, indicating that the nutritional component of rice water has a favorable influence on the vitality of the cells.	[87]
Honey	➢ The nutrients contained, including proteins, vital amino acids, vitamins, and minerals, may nourish and sustain the viability of PDL cells in honey milk with an extended shelf life.	[88]

## Data Availability

No associated data marked.
